# Nonalcoholic Fatty Liver Disease-Related Chronic Kidney Disease

**DOI:** 10.1155/2020/6630296

**Published:** 2020-12-29

**Authors:** Camelia Cojocariu, Ana-Maria Singeap, Irina Girleanu, Stefan Chiriac, Cristina M. Muzica, Catalin Victor Sfarti, Tudor Cuciureanu, Laura Huiban, Carol Stanciu, Anca Trifan

**Affiliations:** ^1^Department of Gastroenterology, Grigore T. Popa University of Medicine and Pharmacy, Iasi 700115, Romania; ^2^St. Spiridon Emergency Hospital, Iasi 700115, Romania

## Abstract

Nonalcoholic fatty liver disease has become the main concern of hepatologists around the world and the main research topic for identifying effective and safe therapy. Advances in the treatment of chronic viral hepatitis in recent years have opened the way towards reducing mortality in patients with chronic liver disease. This goal has not yet been reached, as the burden of chronic liver disease remains a future major health problem as the incidence of the nonalcoholic fatty liver disease continues to rise. The proportion of patients with liver cirrhosis and those with hepatocellular carcinoma due to nonalcoholic liver disease on the liver transplant waiting list has increased in the last years. The upward trend in the incidence and prevalence of the disease in recent decades raises concern over a possible global epidemic, especially as the disease is still underestimated and underdiagnosed. Chronic kidney disease presented an increase in incidence and prevalence during the last years, and it has been associated not only with increased morbidity and mortality but also with high costs for the health system. During the last decade, several studies have shown the association between nonalcoholic fatty disease and chronic kidney disease, two major worldwide health problems.

## 1. Introduction

Nonalcoholic fatty liver disease (NAFLD) and chronic kidney disease (CKD) are worldwide public health problems, due to their increasing prevalence, poor outcomes, and health care burden [1–5]. Nonalcoholic fatty liver disease is the most common etiology of chronic liver disease worldwide, especially in developing countries.

NAFLD covers a wide range of diseases from benign steatosis (fat accumulation in >5% of hepatocytes, especially macrovesicular, without inflammation or fibrosis) to nonalcoholic steatohepatitis (NASH) which is characterized by liver inflammation with high potential to progress to advanced fibrosis, liver cirrhosis, and hepatocellular carcinoma.

In a recent analysis of the Third National Health and Nutrition Survey database (including ∼11,700 American subjects), the moderate to advanced stages of CKD in patients with ultrasound-detected NAFLD were independently associated with increased all-cause mortality over a mean follow-up period of 19 years [6].

During the last decade, several studies have shown the association between NAFLD and CKD, regardless of the presence or not of known risk factors for diseases such as obesity, hypertension, type 2 diabetes mellitus (T2DM), or metabolic syndrome [7–10].

CKD has high rates in patients with NAFLD, ranging between 20% and 50% compared to 5% and 25% in those without [5, 11–14].

## 2. Chronic Kidney Disease: Definition and Staging

CKD is defined as either decreased estimated glomerular filtration rate (eGFR) (<60 ml/min/1.73^2^) and/or abnormal albuminuria and/or overt proteinuria for 3 or more months [5, 7, 15, 16]. Kidney failure is defined as an eGFR of less than 15 mL/min per 1.73 m^2^ or the need for treatment with dialysis/transplantation [15]. The most common tests for CKD diagnosis include eGFR and urinary albumin-to-creatinine ratio (ACR: normal value, <30 mg/g), the essentially diagnostic tools used in classification of CKD patients into five stages [5, 7]. Based on GFR levels, CKD is classified into five stages: stage 1, GFR more than 90 mL/min per 1.73 m^2^; stage 2, 60–89 mL/min per 1.73 m^2^; stage 3, 30–59 mL/min per 1.73 m^2^ (stage 3a, 45–59 mL/min per 1.73 m^2^; stage 3b, 30–44 mL/min per 1.73 m^2^); stage 4, 15–29 mL/min per 1.73 m^2^; and stage 5, GFR less than 15 mL/min per 1.73 m^2^ [16, 17].

## 3. NAFLD and CKD: Epidemiological Data

A large meta-analysis including nearly 64,000 participants in 20 cross-sectional studies and 13 longitudinal studies showed that NAFLD was associated with an approximately 2-fold increase in both prevalence (OR, 2.12; 95% CI, 1.69 to 2.66) and incidence of CKD (HR, 1.79; 95% CI, 1.65 to 1.95) [17]. The NAFLD was most often assessed using noninvasive methods (scoring systems for fibrosis evaluation and ultrasonography) and only in a few cases by liver biopsy.

Some data suggest that the degree of renal impairment is correlated with the histological severity of NASH, the progressive type of NAFLD, and the hepatic fibrosis stage [14]. In the same meta-analysis, NASH was associated with a higher prevalence and incidence of CKD than simple hepatic steatosis, and advanced fibrosis was associated with a higher prevalence (OR, 5.20; 95% CI, 3.14 to 8.61) and incidence (HR, 3.29; 95% CI, 2.30 to 4.71) of CKD than nonadvanced fibrosis [17]. An Italian study on 570 White subjects found that patients with high risk for developing liver fibrosis had a 5.1-fold increased risk of developing CKD compared with low-risk patients (OR: 5.1; 95% CI = 1.13–23.28; *p*=0.03), while intermediate-risk subjects had a 3-fold increased risk of developing liver fibrosis and had 3 times increased risk of developing CKD compared to low-risk patients (OR: 3.01, 95% CI = 0.87–10.32; *p*=0.07) [18].

In a recent longitudinal study, Jang et al. showed that NAFLD is an independent risk factor associated with the progression of CKD. The risk of CKD progression was higher in patients with advanced NAFLD (probably associated with a significant/advanced hepatic fibrosis score) and in those with lower eGFR with/without proteinuria [19].

Similar results were reported by Mantovani in a systematic review and meta-analysis (9 observational cohort studies including approximately 96,500 middle-aged individuals of predominantly Asian descent, over a median follow-up period of 5.2 years). The authors found a 40% increase in the long-term risk of incident CKD (random-effect HR, 1.37; 95% CI, 1.20–1.53; *I*^2^ = 33.5%) correlated with the severity of liver fibrosis; they showed that the risk of CKD in NAFLD patients remained significant even after adjustment for age, sex, obesity, hypertension, smoking, T2DM, baseline eGFR, or medications [20].

Few data suggest that lifestyle modification over a year in patients with biopsy-proven NASH leads to the histologic resolution of NASH and improvement in liver fibrosis stage independently associated with improvement in renal function (increase in eGFR values) [21].

Most of the cohort studies included in meta-analyses are reported in Asian countries, where large populations undergo regular health check-up programs, besides having different genetic, dietary factors, and adipose tissue distributions [5]. The discrepancy between eastern and western populations regarding the NAFLD-CKD relationship has been found in previous studies. While the National Health and Nutrition Examination Survey (NHANES) study of 11,469 US adults showed no increased risk of CKD in patients with ultrasonography diagnosed NAFLD, after correcting for the presence of the metabolic syndrome [12], a large prospective cohort study of 8329 Korean men without T2DM, hypertension, or CKD at baseline followed up for 4 years showed that patients with NAFLD had a significantly higher risk of developing CKD, after correcting for the same risk factors [9, 22].

The relationship between NAFLD and CKD is still poorly understood, and the mechanism relating NAFLD with renal dysfunction to date is yet unknown. Moreover, as in patients with T2DM, the link between NAFLD and CKD is bidirectional, so that kidney damage *per* se subsequently contributes to the progression of liver damage [23].

Many studies suggest that NAFLD and CKD share common pathogenetic mechanisms: oxidative stress, impaired regulation of the renin-angiotensin system, and alterations in the gut microbiota [24].

The currently available data suggest that NAFLD could be a driver force for the development and progression of CKD, rather than a marker of CKD [5].

## 4. NAFLD and CKD: Clinical Approach

In clinical practice, renal function should be evaluated and monitored in all patients with NAFLD as in patients with liver cirrhosis. In a large meta-analysis, Musso et al. suggest that patients with NAFLD should be screened for CKD even in the absence of other classical risk factors, and this is especially recommended if NASH and/or advanced fibrosis are suspected [25].

Although there are no guidelines, surveillance protocols for CKD in patients with NAFLD, it is crucial to detect early renal impairment in these patients to prevent CKD progression, minimize complications, and improve survival [7, 26].

Clinicians should therefore identify CKD stage ≤3; all CKD above stage 3 (abnormal albuminuria (ACR >−30 mg/g) or overt proteinuria, urine sediment abnormalities, and eGFR <60 ml/min/1.73 m^2^ are associated with a high or very high risk of disease progression [5]. Armstrong et al. proposed that the annual screening in patients with NAFLD for CKD by eGFR and microalbuminuria could detect early renal impairment in patients with NAFLD [27], and we consider that, in all new patients diagnosed with NAFLD, the renal function assessment is mandatory, and all medications that could affect kidney function in patients with NAFLD must be evaluated to allow drug-dosage adjustment [28].
